# Supplementary material for: NFYA promotes the anti-tumor effects of gluconeogenesis in hepatocellular carcinoma through the regulation of PCK1 expression
Source: Front Cell Dev Biol. 2022 Aug 25;10:983599. doi: 10.3389/fcell.2022.983599 (PMC9452718; doi:10.3389/fcell.2022.983599)
Supplement: Supplementary file 1 [file DataSheet1.pdf]

Supplementary Material

Supplementary Table S1 Primer sequences for qRT-PCR analyses.

**qRT-PCR primer sequences**	
mouse Actb forward primer	5'- GATCTGGCACCACACCTTCT -3'
mouse Actb reverse primer	5'- GGGGTGTTGAAGGTCTCAAA -3'
mouse Nfyav1 forward primer	5'- AAGTCCAGACCCTCCAGGTAGT -3'
mouse Nfyav1 reverse primer	5'- GATGGGTTGGCCTGTTGAT -3'
mouse Nfyav2 forward primer	5'- GCCATGGAGCAGTATACGACA -3'
mouse Nfyav2 reverse primer	5'- CCTGGACCTGCTGCTGAA -3'
mouse Pck1 forward primer	5'- CCTTTGGAAGCGGATATGGT -3'
mouse Pck1 reverse primer	5'- TTGCCTTCGGGGTTAGTTATG -3'
mouse G6pc forward primer	5'- ACTGTGGGCATCAATCTCCTCT -3'
mouse G6pc reverse primer	5'- GGGCGTTGTCCAAACAGAA -3'
human ACTB forward primer	5'- ACCAACTGGGACGACATGGAGAAA -3'
human ACTB reverse primer	5'- TAGCACAGCCTGGATAGCAACGTA -3'
human PCK1 forward primer	5'- GACATTGCCTGGATGAAGTTTG -3'
human PCK1 reverse primer	5'- TTCTTCTGGATGGTCTTGATGG -3'
human SLC1A5 forward primer	5'- TCCGCTTCTTCAACTCCTTCA -3'
human SLC1A5 reverse primer	5'- AAACCCACATCCTCCATCTCC -3'
human GLS forward primer	5'- TTCTCAGGGCAGTTTGCTTTC -3'
human GLS reverse primer	5'- TTGCCCATCTTATCCAGAGGA -3'
human GLUD1 forward primer	5'- AAGGCAAAGCCCTATGAAGGA -3'
human GLUD1 reverse primer	5'- CATTGGCACCTTCAGCAATG -3'
human GOT2 forward primer	5'- ACCGGGATGATAATGGAAAGC -3'
human GOT2 reverse primer	5'- CTTGCAAAATTCAGCCAGTCC -3'
human OGDH forward primer	5'- GCAGATGTGCAACGATGACC -3'
human OGDH reverse primer	5'- TGGAAGAAGTTGCCAGGAGTG -3'
human PCK1 forward primer for CUT&RUN assay	5'- GGTGCATCCTTCCCATGAAC -3'
human PCK1 reverse primer for CUT&RUN assay	5'- CTGGTTGGCAAAACACCACA -3'

Tsujimoto and Ito *et al.* Supplementary Figure S1

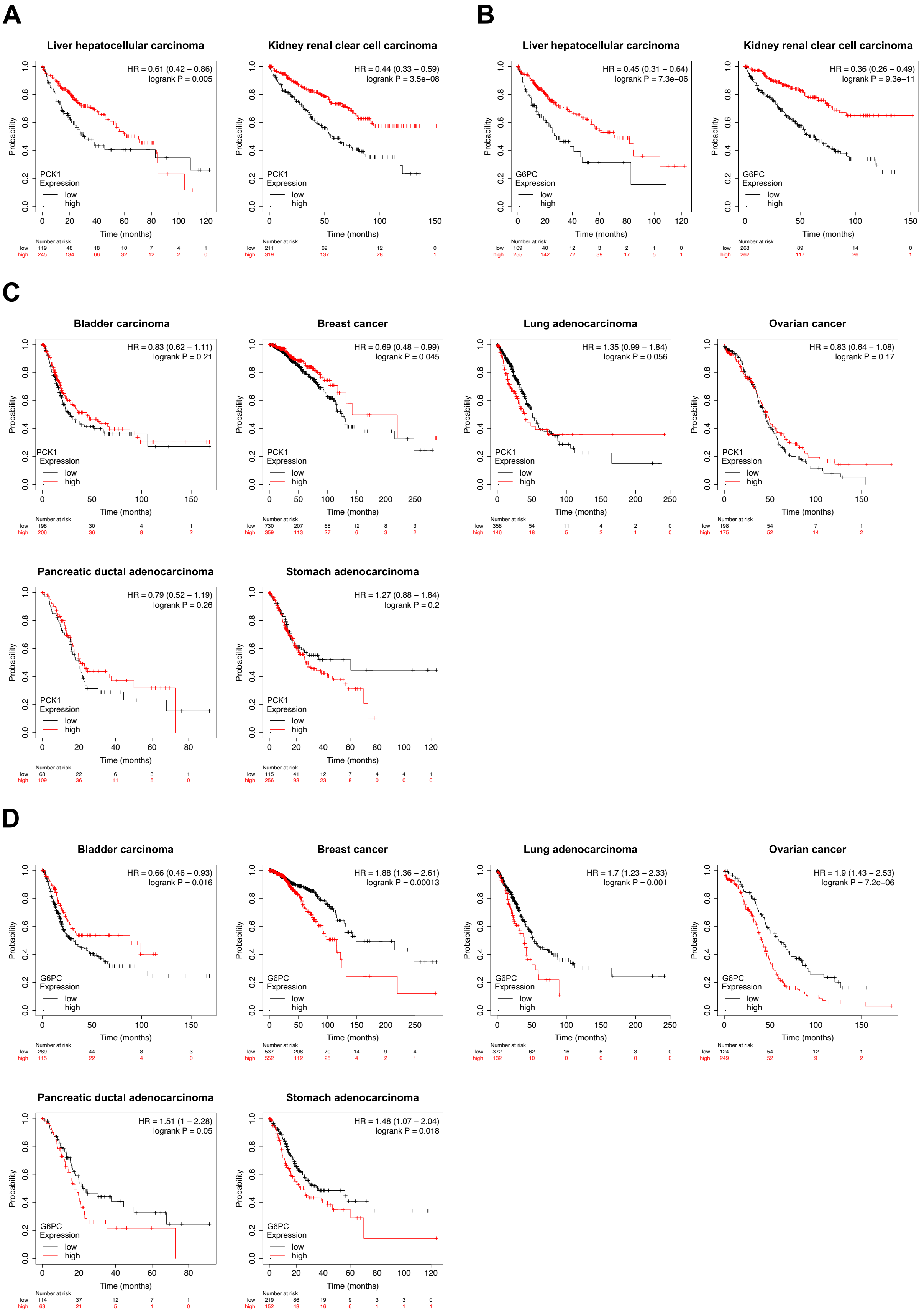

Supplementary Figure S1. The expression of PCK1 and G6PC predicts better patient survival only for gluconeogenic organ cancer. Kaplan-Meier plots of overall survival of patients with gluconeogenic organ cancer (A; for PCK1, B; for G6PC) and non-gluconeogenic organ cancer (C; for PCK1, D; for G6PC). Data were obtained from the Kaplan-Meier plotter online tool. The hazard ratio (HR) and respective log-rank p-values identifying the high-expression group (red) and the low-expression group (black) are shown.

Tsujimoto and Ito *et al.* Supplementary Figure S2

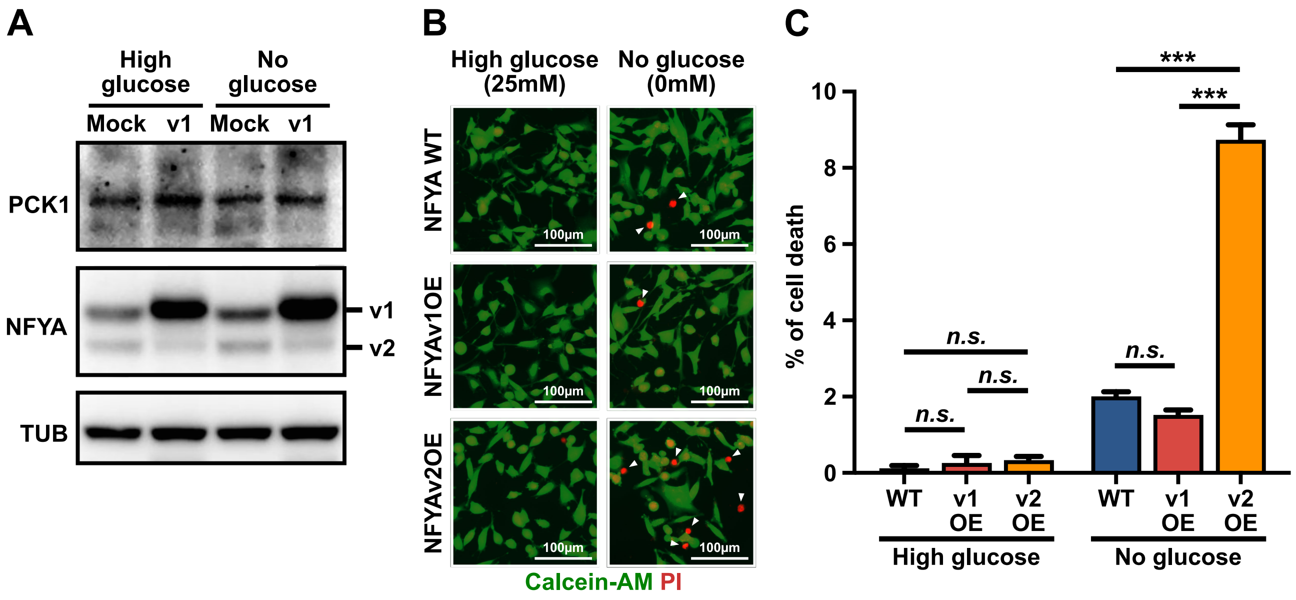

Supplementary Figure S2. NFYAv2 enhances the anti-tumor effects of gluconeogenesis. **(A)** Western blot analysis of the expression levels of PCK1 and NFYA in NFYAv1OE SK-Hep1 cells under normal or glucose deprivation conditions after 10 hours culture. **(B)** Representative fluorescence images of living cells (green) detected with Calcein-AM and dead cells (red) detected with Propidium iodide (PI) in wild-type, NFYAv1OE, and NFYAv2OE SK-Hep1 cells under normal or glucose deprivation conditions after 10 hours culture. **(C)** The bar graph shows the percentage of dead cells among the more than 1,500 cells counted each condition. Data are shown as mean and SEM. Unpaired two-tailed t-test was performed. (n.s.) not significant; (***) P<0.001.

Tsujimoto and Ito *et al.* Supplementary Figure S3

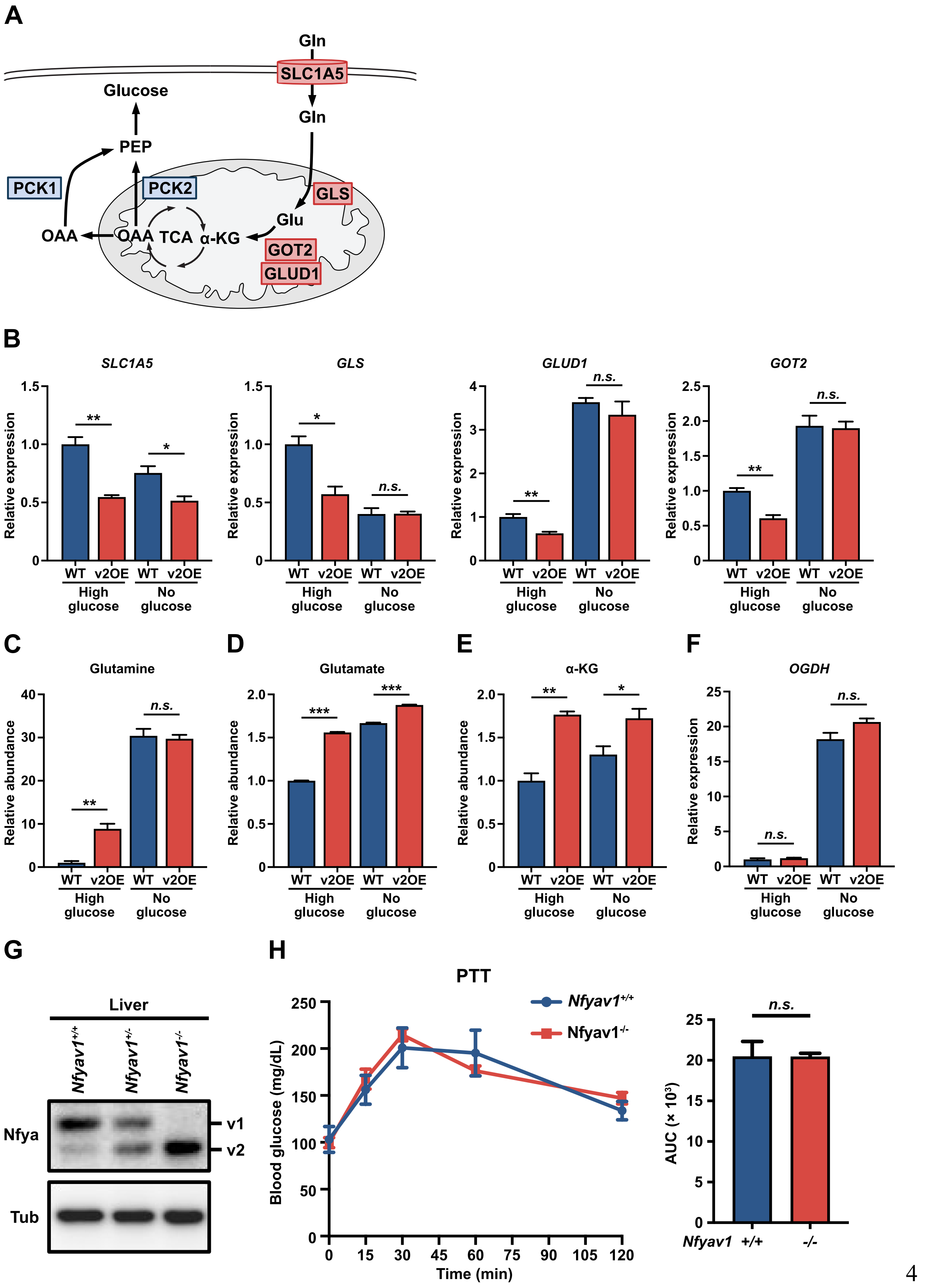

Supplementary Figure S3. Glutaminolysis is normal in NFYAv2OE SK-Hep1 cells. **(A)** A diagram illustrating the reaction of glutaminolysis and gluconeogenesis. **(B)** qRT-PCR analysis of the expression levels of SLC1A5, GLS, GLUD1, and GOT2 in wild-type and NFYAv2OE SK-Hep1 cells under normal or glucose deprivation conditions after 5 hours of culture. **(C-E)** The intracellular glutamine (C), glutamate (D), and a-KG (E) levels in wild-type and NFYAv2OE SK-Hep1 cells under normal or glucose deprivation conditions after 5 hours of culture. **(F)** qRT-PCR analysis of the expression levels of OGDH in wild-type and NFYAv2OE SK-Hep1 cells under normal or glucose deprivation conditions after 5 hours of culture. **(G)** Western blot analysis of Nfya protein levels in the liver tissue of *Nfyav1*$^{+/+}$, *Nfyav1*$^{+/-}$, and *Nfyav1*$^{-/-}$ mice. **(H)** Pyruvate tolerance test (PTT) in *Nfyav1*$^{+/+}$ (n=5) and *Nfyav1*$^{-/-}$ (n=4) mice. A bar graph shows the area under curve. All data are shown as mean and SEM. Unpaired two-tailed t-test was performed. (n.s.) not significant; (*) P<0.05; (**) P<0.01; (***) P<0.001.

Tsujimoto and Ito *et al.* Supplementary Figure S4

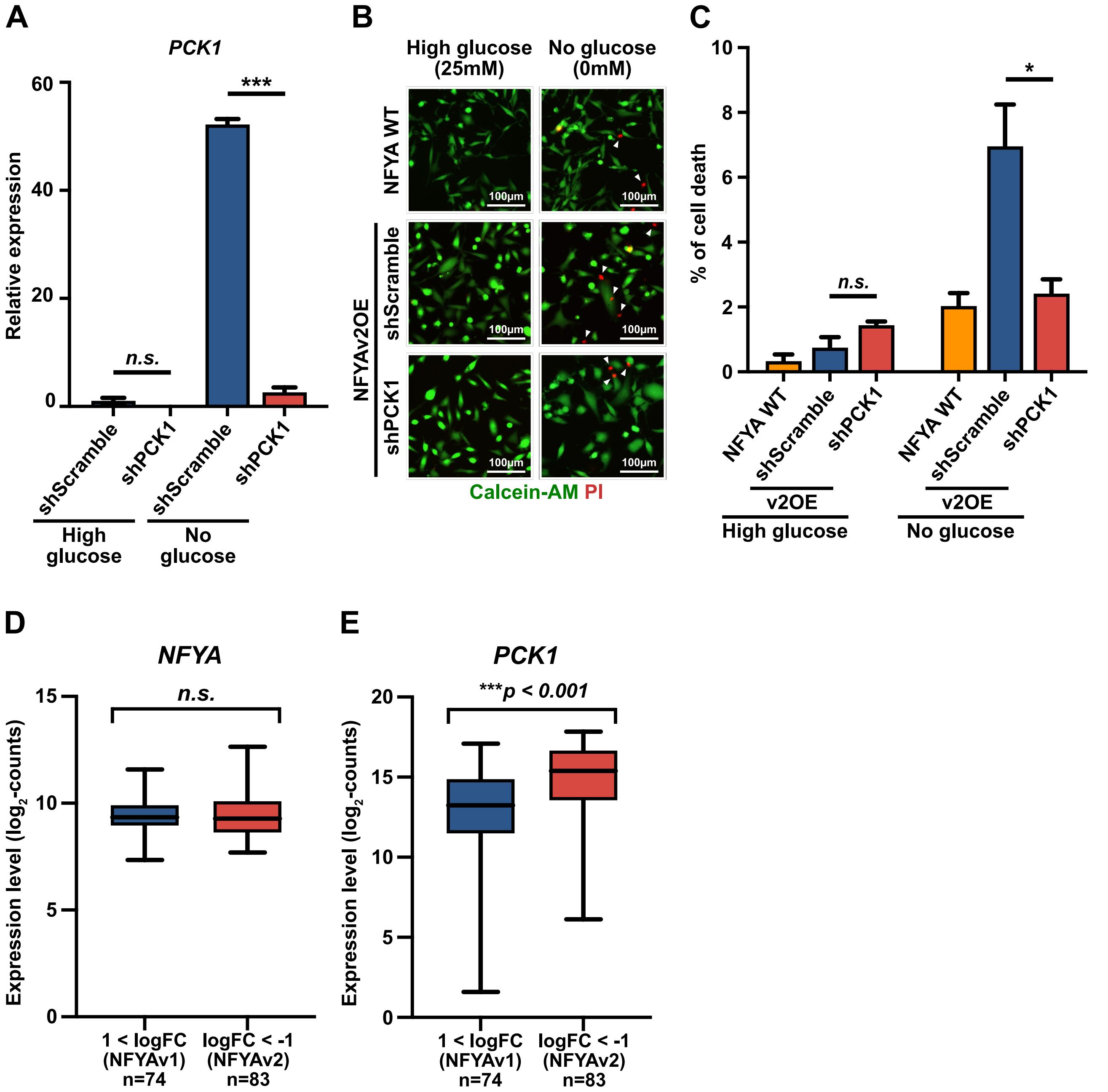

Supplementary Figure 4. NFYAv2 enhances gluconeogenesis by transcriptional activation of PCK1. **(A)** qRT-PCR analysis to validate the knockdown of *PCK1* gene expression in NFYAv2OE SK-Hep1 cells under normal or glucose deprivation conditions after 5 hours of culture. Data are shown as mean and SEM. Unpaired two-tailed t-test was performed. (n.s.) not significant; (***) P<0.001. **(B)** Representative fluorescence images of living cells (green) detected with Calcein-AM and dead cells (red) detected with Propidium iodide (PI) in wild-type and NFYAv2OE SK-Hep1 cells knocked down PCK1 gene expression under normal or glucose deprivation conditions after 10 hours culture. **(C)** The bar graph shows the percentage of dead cells among the more than 600 cells counted

each condition. Data are shown as mean and SEM. Unpaired two-tailed t-test was performed. (n.s.) not significant; (*) P<0.05. **(D, E)** Using RNA-seq data from TCGA, 297 human hepatocellular carcinomas were classified into two groups according to logFC of VIM/CDH1: one group predominantly expressing NFYAv1 (1 < logFC: n=74) and another group predominantly expressing NFYAv2 (logFC < -1: n=83). The expression levels (log2 counts) of NFYA (D) and PCK1 (E) in each group were calculated. Unpaired two-tailed t-test was performed. (n.s.) not significant; (***) P<0.001.
